# Supplementary material for: Coping with Workplace Incivility in Hospital Teams: How Does Team Mindfulness Influence Prevention- and Promotion-Focused Emotional Coping?
Source: Int J Environ Res Public Health. 2022 Dec 3;19(23):16209. doi: 10.3390/ijerph192316209 (PMC9738836; doi:10.3390/ijerph192316209)
Supplement: Supplementary file 1 [file ijerph-19-16209-s001.zip › ijerph-2021717-supplementary.pdf]

Table S1: Venting Multilevel Results

Level and variable	Model					
	**1. Null**	**2. Fixed effects of controls**	**3. Fixed effect of incivility**	**4. Fixed effect of team mindfulness**	**5. Random effect of incivility**	**6. Cross-level interaction**
**Level 1**						
Age		.02 (.01)	.02 (.01)	.02 (.01)	.02 (.01)	.01 (.01)
Gender		.42 (.22)	.45* (.22)	.45* (.21)	.43* (.21)	.42* (.21)
Incivility			.20** (.07)	.20** (.07)		
**Level 2**						
Team size		.01 (.02)	.01 (.02)	.01 (.02)	.01 (.02)	.01 (.02)
Team mindfulness				-.74** (.20)	-.72** (.21)	-.74** (.20)
**Cross-level interaction**						
Incivility Team mindfulness						-.47* (.21)
ICC	.10					
–2 log likelihood	1548.11	1507.06	1497.85	1485.68	1485.42	1479.68
Number of estimated parameters	3	6	7	8	10	11

Values in parentheses are standard errors; *p < .05. **p < .01.

Table S2: Behavioral Disengagement Multilevel Results

Level and variable	Model					
	**1. Null**	**2. Fixed effects of controls**	**3. Fixed effect of incivility**	**4. Fixed effect of team mindfulness**	**5. Random effect of incivility**	**6. Cross-level interaction**
**Level 1**						
Age		.01 (.01)	.01 (.01)	.01 (.01)	.01 (.01)	.01 (.01)
Gender		.00 (.21)	.04 (.21)	.04 (.21)	.02 (.21)	.02 (.21)
Incivility			.27** (.06)	.27** (.06)		
**Level 2**						
Team size		-.03 (.03)	-.03 (.03)	-.03 (.02)	-.03 (.02)	-.03 (.02)
Team mindfulness				-.53* (.21)	-.49* (.21)	-.52* (.21)
**Cross-level interaction**						
Incivility Team mindfulness						-.23 (.20)
ICC	.15					
–2 log likelihood	1485.76	1454.95	1435.43	1429.60	1425.33	1424.05
Number of estimated parameters	3	6	7	8	10	11

Values in parentheses are standard errors; *p < .05. **p < .01.

Table S3: Denial Multilevel Results

Level and variable	Model					
	**1. Null**	**2. Fixed effects of controls**	**3. Fixed effect of incivility**	**4. Fixed effect of team mindfulness**	**5. Random effect of incivility**	**6. Cross-level interaction**
**Level 1**						
Age		.01 (.01)	.01 (.01)	.01 (.01)	.01 (.01)	.01 (.01)
Gender		-.58** (.22)	-.55** (.22)	-.57** (.22)	-.61** (.22)	-.61** (.22)
Incivility			.17** (.06)	.17** (.06)		
**Level 2**						
Team size		-.02 (.03)	-.02 (.03)	-.02 (.03)	-.02 (.03)	-.02 (.03)
Team mindfulness				-.46* (.22)	-.46* (.22)	-.46* (.22)
**Cross-level interaction**						
Incivility Team mindfulness						-.25 (.22)
ICC	.19					
–2 log likelihood	1516.70	1477.19	1470.03	1465.85	1462.10	1460.82
Number of estimated parameters	3	6	7	8	10	11

Values in parentheses are standard errors; *p < .05. **p < .01.

Table S4: Acceptance Multilevel Results

	Model					
**Level and variable**	**1. Null**	**2. Fixed effects of controls**	**3. Fixed effect of incivility**	**4. Fixed effect of team mindfulness**	**5. Random effect of incivility**	**6. Cross-level interaction**
**Level 1**						
Age		-.01 (.01)	-.01 (.01)	.01 (.01)	.01 (.01)	.01 (.01)
Gender		-.22 (.16)	-.22 (.16)	-.21 (.16)	-.23 (.16)	-.23 (.16)
Incivility			.02 (.05)	.02 (.05)		
**Level 2**						
Team size		.03 (.02)	.03 (.02)	.04 (.02)	.03 (.02)	.03 (.02)
Team mindfulness				-.19 (.15)	-.22 (.15)	-.20 (.15)
**Cross-level interaction**						
Incivility Team mindfulness						-.15 (.15)
ICC	.05					
–2 log likelihood	1265.63	1232.78	1232.62	1230.93	1229.65	1228.64
Number of estimated parameters	3	6	7	8	10	11

Values in parentheses are standard errors; *p < .05. **p < .01.

Table S5: Positive Reframing Multilevel Results

Level and variable	Model					
	**1. Null**	**2. Fixed effects of controls**	**3. Fixed effect of incivility**	**4. Fixed effect of team mindfulness**	**5. Random effect of incivility**	**6. Cross-level interaction**
**Level 1**						
Age		.01 (.01)	.01 (.01)	.01 (.01)	.01 (.01)	.01 (.01)
Gender		-.16 (.15)	-.16 (.15)	-.16 (.15)	-.16 (.15)	-.16 (.15)
Incivility			-.01 (.04)	-.01 (.04)		
**Level 2**						
Team size		.03 (.02)	.03 (.02)	.03 (.02)	.03 (.02)	.03 (.02)
Team mindfulness				-.20 (.16)	-.21 (.16)	-.20 (.16)
**Cross-level interaction**						
Incivility Team mindfulness						-.04 (.14)
ICC	.17					
–2 log likelihood	1181.52	1156.38	1156.36	1154.79	1153.82	1153.75
Number of estimated parameters	3	6	7	8	10	11

Values in parentheses are standard errors; *p < .05. **p < .01.
